# Postoperative Analgesics Score as a Predictor of Chronic Postoperative Inguinal Pain After Inguinal Hernia Repair: Lessons Learned From a Retrospective Analysis

**DOI:** 10.1007/s00268-023-07074-6

**Published:** 2023-05-29

**Authors:** A. Widder, L. Reese, J. F. Lock, A. Wiegering, C.-T. Germer, G.-K. Kindl, H. L. Rittner, U. Dietz, J. Doerfer, N. Schlegel, M. Meir

**Affiliations:** 1 Department of General, Visceral, Transplantation, Vascular and Pediatric Surgery; Center of Operative Medicine (ZOM), University Hospital of Würzburg, Würzburg, Germany; 2 Center for Interdisciplinary Pain Medicine, Department of Anesthesiology, Intensive Care Medicine, Emergency Medicine and Pain Therapy, University Hospital of Wuerzburg, Würzburg, Germany; 3 Department of Visceral, Vascular and Thoracic Surgery, Cantonal Hospital Olten (soH), Olten, Switzerland

## Abstract

**Background:**

Chronic postoperative inguinal pain (CPIP) is a common complication after inguinal hernia surgery and occurs in up to 10–14% of cases. CPIP has a significant impact on daily life, work ability and thus compromises quality of life. The aim of this retrospective study was an in-depth analysis of patients undergoing inguinal hernia repair to further refine the prediction of the onset of CPIP reliably.

**Methods:**

A single center retrospective analysis of patients with who underwent open or minimally invasive inguinal hernia repair from 2016 to 2021 was carried out. Complication rates, detailed analysis of postoperative pain medication and quality of life using the EuraHS Quality of Life questionnaire were assessed.

**Results:**

Out of 596 consecutive procedures, 344 patients were included in detailed analyses. While patient cohorts were different in terms of age and co-morbidities, and the prevalence of CPIP was 12.2% without differences between the surgical procedures (Lichtenstein: 12.8%; TEP 10.9%; TAPP 13.5%). Postoperative pain was evaluated using a newly developed analgesic score. Patients who developed CPIP later had a significant higher consumption of analgesics at discharge (*p* = 0.016). As additional risk factors for CPIP younger patient age and postoperative complications were identified.

**Conclusion:**

The prospective use of the analgesic score established here could be helpful to identify patients that are at risk to develop CPIP. These patients could benefit from a structured follow-up to allow early therapeutic intervention to prevent chronification and restore the quality of life.

## Introduction

Surgical treatment of inguinal hernia is one of the most frequent general surgical procedures with more than 20 million patients annually. Both, open and minimally-invasive procedures exist, although, apart from the recommendation to use a mesh, there is no standard repair technique [1]. The open procedure “Lichtenstein” and two endoscopic techniques, extraperitoneal (TEP) or transperitoneal (TAPP), are proposed as the optimal repair techniques [1]. Some studies have associated endoscopic techniques with shorter recovery time and lower risk of chronic postoperative inguinal pain (CPIP), as well as a higher risk of recurrence in hernias [2, 3]. However, analyses also reported a similar chronic pain rate and the same recurrence and complication rate (hematoma, seroma, wound infections) after open and minimal-invasive surgical procedures [4, 5]. Despite all the advances in surgery, countless studies, and the evolution of hernia meshes (e.g., in terms of pore sizes and amount of material), this significant CPIP incidence has not been reduced in the last 30 years, hernia surgery is treading water. CPIP affects up to 14% of patients. CPIP is defined as pain (≥3 in a visual analogue or numeric rating scale (V/NRS) from 0–10 for pain) lasting at least 3 months postoperatively that interferes with daily activities, is perceived as bothersome and sometimes has neuropathic components [1, 6]. Although CPIP resolves within one year postoperatively in up to 70% of cases, 30% of patients suffer from it permanently [7]. Young age, female gender, severe perioperative pain, recurrent hernia and open hernia repair have previously been reported as risk factors for the development of CPIP [8]. However, high early perioperative pain intensity has not been clearly defined or quantified in previous publications [1, 9–11] although this represents a risk factor that can be modified by adequate pain medication. On the other hand, it is unclear whether the need of pain medication to ameliorate perioperative pain itself is a risk factor or whether the adequate perioperative pain management reduces the risk of developing CPIP. Therefore, we performed a detailed retrospective analysis of patients undergoing inguinal hernia repair with a specific focus on perioperative pain management to further identify the aspect of perioperative pain management as a risk factor for CPIP.

## Material and methods

### Study design

A retrospective single-center analysis of all consecutive patients who underwent inguinal hernia repair at the Department of Surgery at Würzburg University Hospital and gave their informed consent between January 1, 2016, and December 31, 2021, was carried out. The study was approved by the local ethics committee (No. 242/17).

#### Data acquisition

Clinical data (patient baseline characteristics: age, sex, symptoms, ASA score; surgery: type of repair, operation time; complications according to the Clavien-Dindo-classification (CDC), need for perioperative pain medication; duration of hospital stay) of patients were retrieved from the local prospectively recorded database. In addition a postoperative survey of all patients using the EuraHS-Quality of life (Qol) questionnaire [12, 13] was carried out. In this questionnaire the assessment includes an assessment on postoperative pain, restriction in daily activities and cosmetic comfort after the operation. Both minimally invasive procedures, TEP and TAPP and the open procedure according to Lichtenstein were included in the evaluation.

### Definition of CPIP in this study

We adopted the CPIP definition according to the current EuraHS guidelines as pain (≥3 in a V/NRS from 0–10; 0 for no pain and 10 for maximal pain) lasting more than 3 months postoperatively in the EuraHS-Qol questionnaire [14].

### Establishment of the analgesics score

**Fig. 1:**
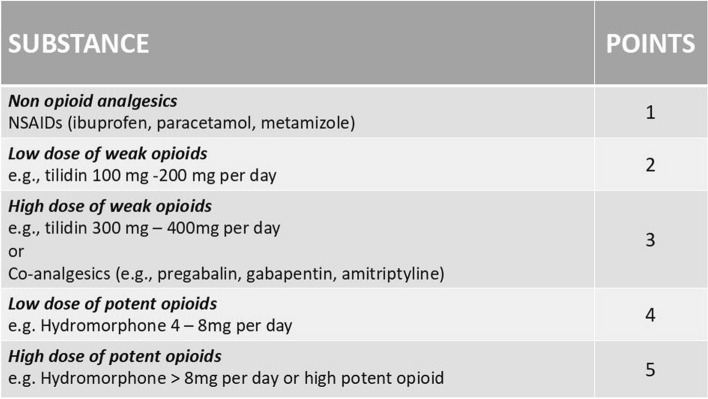
Analgesics score 352 × 198 mm (96 × 96 DPI)

Mean postoperative pain is a composite of the pain intensity on the V/NRS and the amount and potency of analgesics taken. To enable the comparison of postoperative pain an analgesic score was established. This was based on the need of postoperative pain medication at the day of discharge. Postoperative analgesic therapy was adjusted based on standard procedures until the patients were pain-free at rest (V/NRS <3). This was documented at discharge. In order to quantify pain postoperatively, the analgesics taken at the timepoint of hospital discharge were defined according to their potency using an ascending point scale based on the WHO analgesics ladder and the dose taken. With this scale an analgesic score could be calculated (Fig. 1). Each substance (amount) was assigned a scoring. To reduce the bias by prescribed analgesics independent of their target—groin pain or other sites of pain—these were subtracted from the score. The pain score is the sum of all analgesic medications at hospital discharge.

### Statistical analysis

Statistical analysis was performed using IBM SPSS 28.0 (IBM SPSS, Armonk, New York, USA). Differences between groups were calculated using Welch’s test and Chi^2^-test as well as single factor variance and analysis of covariance or as repeated measure ANOVA. In the case of multiple *T*-tests, a post-hoc test was used by means of Bonferroni correction to detect individual comparisons between the groups without risking an alpha error. Significance was set at *p* < 0.05. Descriptive analyses included mean (MV), minimum (min) and maximum (max) values or standard deviation (indicated by ±).

## Results

### Characterization of the study population

**Fig. 2:**
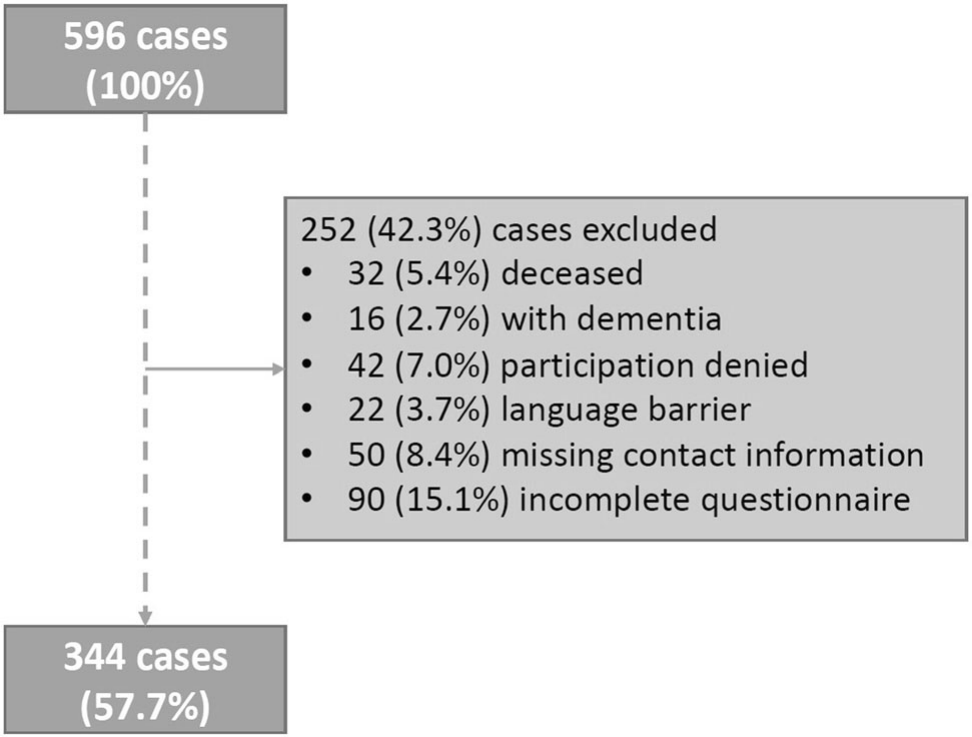
Exclusion criteria 301 × 222 mm (96 × 96 DPI)

A total of 596 patients with groin hernia repair were identified in the retrospective evaluation of our prospectively managed database. As presented in Fig. 2, 252 (42.3%) patients were excluded from the data analysis: 32 patient cases were deceased, 16 patient cases suffered from pre-existing or newly diagnosed dementia, in 42 cases participation in the study was refused, in 22 patients a language barrier made accurate data collection impossible. Similarly, 50 patient cases could not be contacted due to changes in contact and address data, and 90 cases did not respond with a complete questionnaire. Overall, 344 (57.7%) patients were analyzed using the EuraHS-QoL questionnaire.

The 344 patients were divided into three groups depending on the surgical technique: Lichtenstein as TEP and TAPP. 86.6% of the patients were male. Patients who received Lichtenstein surgery were significantly older (68.2 *y*; range 22–89) than patients who received TAPP (54.4 *y*; range 18–89) or TEP (53.2 *y*; range 19–82) (*p* = 0.001). If TEP was performed, the BMI was significantly lower than with the TAPP or the Lichtenstein (*p* = 0.035). All three surgical procedures differed significantly in the ASA classification of the operated patients (Lichtenstein: 2.4 ± 0.58; TEP 1.7 ± 0.51; TAPP 2.0 ± 0.53). Patients who underwent Lichtenstein surgery had significantly more malignancies in their history and had a significantly higher rate of anticoagulant medication. In our cohort 27.1% of the Lichtenstein patients took warfarin or non-Vitamin-K-antagonist Oral Anticoagulants compared to 5% in TAPP or TEP (Table 1).

**Table 1:** Baseline characteristics

Characteristics *n* (%)	All = 344 (100%)	Lichtenstein = 141 (41%)	TEP = 129 (37.5%)	TAPP = 74 (21,5%)	*p*-value
Sex					.001
Male *n* (%)	298 (86.6)	125 (88.7)	129 (100)	44 (59.5)	
Female *n* (%)	46(13.4)	16 (11.3)	0	30 (40.5)	
Age at operation [years] median (range)	59.6 (18–89)	68.2 (22–89)	53.2 (19–82)	54.4 (18–89)	.001
BMI [kg/m^2^] median (range)	25.5 (16.7–49.6)	26.1 (18.1–49.6)	24.9 (16.7–35.5)	25.4 (17.5–40.1)	.035
ASA classification median (range)	2.1 (1–4)	2.4 (1–4)	1.7 (1–3)	2.0 (1–3)	.001
Hernia recurrence *n* (%)	28 (8.1)	6 (4.3)	13 (10.1)	9 (12.2)	.078
Malignoma *n* (%)	48 (14)	38 (27)	5 (3.9)	5 (6.8)	.001
Smoking *n* (%)	75 (21.8)	36 (25.5)	22 (17.1)	17 (23)	.233
Other Neurologic diseases (%)	1 (1.6)	1 (2.9)	1 (2.9)	0	.009
Immunsupression *n* (%)	20 (5.8)	11 (7.8)	5 (3.9)	4 (5.4)	.382
Chronic use of pain medication *n* (%)	11 (3.2)	6 (4.3)	3 (2.3)	2 (2.7)	.642
Diabetes mellitus *n* (%)	26 (7.6)	16 (11.3)	5 (3.9)	5 (6.8)	.077
DMT1 *n* (%)	1 (0.3)	0	1 (0.8)	0	
DMT2 *n* (%)	25 (7.3)	16 (11.3)	4 (3.1)	5 (6.8)	
Anticoagulation *n* (%)	101 (29.3)	67 (47.1)	20 (15.5)	14 (18.9)	.001
NOAC *n* (%)	29 (8.5)	22 (15.7)	4 (3.1)	3 (4.1)	
Warfarin *n* (%)	19 (5.5)	16 (11.4)	1 (0.8)	2 (2.7)	
ASS *n* (%)	48 (14)	24 (17.1)	15 (11.6)	9 (12.2)	
Clopidogrel *n* (%)	4 (1.2)	4 (2.9)	0	0	

### Similar postoperative outcome after the different procedures

Operation time was significantly longer in patients receiving TAPP (86 min; *p* = 0.001), whereas TEP and Lichtenstein surgery showed no significant difference (both 71 min). Patients receiving Lichtenstein surgery stayed significantly longer in the hospital compared to patients who underwent minimally invasive procedures (Lichtenstein: 2.95 ± 2.1 *d*; TEP 1.22 ± 0.60 *d*; TAPP 1.72 ± 0.87 *d*). Patients who underwent Lichtenstein surgery had significantly more postoperative hematomas (*p* = 0.015) than patients who underwent TEP or TAPP (Table 2). Yet, the complications ≥3 according to the CDC did not show any significant differences between the surgical techniques.

**Table 2:** Postoperative characteristics

Complications*n* (%)	All = 344	Lichtenstein = 141 (41%)	TEP = 129 (37.5%)	TAPP = 74 (21,5%)	*p*-value
Operating time [min] median (range)	74 (30–195)	71 (30–195)	71 (32–134)	86 (43–161)	.001
QoL EuraHS median (range)	5.3 (0–82)	5.9 (0–82)	4.8 (0–77)	5.1 (0–51)	.738
CPIP *n* (%)	42 (12.2)	18 (12.8)	14 (10.9)	10 (13.5)	.827
Days in hospital median (range)	2.04 (1–14)	2.95 (1–14)	1.22 (1–5)	1.72 (1-8)	.001

Characteristics *n* (%)	All = 344	Lichtenstein = 141 (41%)	TEP = 129 (37.5%)	TAPP = 74 (21,5%)	*p*-value
Seroma *n* (%)	12 (3.5)	5 (3.5)	4 (3.1)	3 (4.1)	.937
Hematoma *n* (%)	29 (8.4)	19 (13.5)	5 (3.9)	5 (6.8)	.015
Surgical side infection *n* (%)	3 (0.9)	0	2 (1.6)	1 (1.4)	.346
Clavien Dindo classification *n* (%)					.240
Grade I	24 (7.0)	14 (9.9)	6 (4.7)	4 (5.4)	
Grade II	1 (0.3)	1 (0.7)	0	0	
Grade III *a*	0	0	0	0	
Grade III *b*	5 (1.5)	4 (2.8)	0	1 (1.4)	
Grade IV *a*	1 (0.3)	1 (0.7)	0	0	
Grade IV *b*	0	0	0	0	
Grade V	0	0	0	0	
Clavien Dindo ≥ grade 3 *n* (%)	6 (1.7)	5 (3.5)	0	1 (1.4)	.081

The overall prevalence of CPIP 3 months after surgery in the whole patient cohort was 12.2%. Patients after TAPP had a slightly higher incidence of CPIP than those undergoing TEP or Lichtenstein (Lichtenstein: 12.8%; TEP 10.9%; TAPP 13.5%) (Table 2). However, no significant differences were evident between the surgical procedures.

### Surgical complications and experience of the surgeon as contributing factors for CPIP

To further identify risk factors to develop CPIP, we compared the 42 patients that had developed CPIP with 302 patients that had no CPIP according to the survey. CPIP patients had a significantly lower quality of life in the EuraHS-QoL questionnaire (*p* = 0.001). They were significantly younger (54. 4 ± 16.0 *y* vs. 60.3 ± 15.9 *y*) (*p* =0.024). There was no significant difference in hernia orifice size between the groups. Also a similar number of patients did regularly consume analgesic medication. (Table 3).

**Table 3:** Baseline characteristics of subgroup analyses “no pain” versus “CPIP”

Characteristics *n* (%)	No pain = 302 (87.8%)	CPIP = 42 (12.2%)	*p*-value
Sex			.249
Male *n* (%)	264 (88.6)	34 (11.4)	
Female *n* (%)	38(82.6)	8 (17.4)	
Age at operation [years] median (range)	60.3 (19–89)	54.4 (18–86)	.024
BMI [kg/m^2^] median (range)	25.4 (16.7–40.1)	26.4 (17.5–49.6)	.092
ASA classification median (range)	2.1 (1–4)	2.1 (1–3)	.928
Hernia recurrence *n* (%)	24 (7.9)	4 (9.5)	.726
Malignoma *n* (%)	43 (14.2)	5 (11.9)	.683
Nicotin *n* (%)	61 (20.2)	14 (33.3)	.053
Neurology diagnose *n* (%)	20 (6.6)	4 (9.5)	.489
Immunosupression *n* (%)	19 (6.3)	1 (2.4)	.310
Prior pain medication *n* (%)	8 (2.6)	3 (7.1)	.121
Diabetes mellitus *n* (%)	22 (7.3)	4 (9.5)	.781
DMT1 *n* (%)	1 (0.3)	0	
DMT2 *n* (%)	21 (7)	4 (9.5)	
Neurectomia *n* (%)	22 (7.3)	1 (2.4)	.233
Hernia orifice size *n* (%)			.190
<1.5 cm	40 (13.9)	9 (22.9)	
1.5–3.0 cm	183 (63.8)	26 (65)	
>3 cm	64 (22.3)	5 (12.5)	
Previous operation *n* (%)			.375
Laparoscopic surgery	1 (0.3)	1 (2.4)	
Open surgery	20 (6.6)	3 (7.1)	
Open and laparoscopic surgery	3 (1.0)	0	

Next, the influence of surgical aspects was examined. The operation time had no significant influence on the development of CPIP (“no pain” 73 min versus CPIP 77 min). But, there was a significant difference in postoperative complications ≥ 3 according to CDC (*p* = 0.004): CPIP patients had significantly more postoperative complications like hematoma, surgical site infection or seroma that had to be treated operatively or interventional than patients with no pain (CPIP: 7.1%; no pain 1%)(Table 4).

**Table 4:** Postoperative characteristics “no pain” versus “CPIP”

Characteristics	No pain = 302 (87.8%)	CPIP = 42 (12.2%)	*p*-value
Operating time [min] median (range)	73.6 (30–195)	77.5 (40–174)	.337
QoL EuraHS (range)	1.8 (0–18)	30.4 (7–82)	.001
Days in hospital median (range)	1.97 (1–14)	2.5 (1–9)	.053

Complications *n* (%)	No pain = 302 (87.8%)	CPIP = 42 (12.2%)	*p*-value
Seroma *n* (%)	10 (3.3)	2 (4.8)	.631
Hematoma *n* (%)	24 (7.9)	5 (11.9)	.387
Surgical site infection *n* (%)	2 (0.7)	1 (2.4)	.262
Clavien Dindo classification *n* (%)			.001
Grade I	19 (6.3)	5 (11.9)	
Grade II	0	1 (2.4)	
Grade III *a*	0	0	
Grade III *b*	3 (1.0)	2 (4.8)	
Grade IV *a*	0	1 (2.4)	
Grade IV *b*	0	0	
Grade V	0	0	
Clavien Dindo ≥ grade 3 *n* (%)	3 (1.0)	3 (7.1)	.004

Furthermore, a subgroup analysis of the expert level of the surgeons was carried out to show the correlation between expert level and CPIP as well as the implanted mesh type [15]. For the patient it makes no difference whether a surgical resident, a surgical consultant or an attending surgeon performed the operation. The incidence of CPIP was the same. (Table 5).

**Table 5:** sub-group analysis “expertise”

Expertise *n* (%)	Low* = 36 (10.5%)	Medium* = 33 (9.6%)	High* = 275 (79.9%)	*P*-value
*Pain*				
No pain *n* (%)	32 (88.9)	27 (81.8)	234 (88.4)	.543
Cpip *n* (%)	4 (11.1)	6 (18.2)	32 (11.6)	

### Analgesics score as a predictor of CPIP

**Fig. 3:**
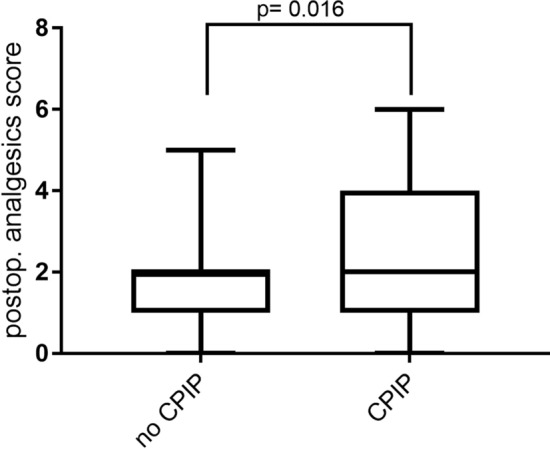
Analgesics Score 92 × 78 mm (300 × 300 DPI)

Using our new analgesic score, patients who eventually developed CPIP consumed significantly more analgesics at the day of discharge (*p* = 0.016) to achieve freedom from pain than patients who did not exhibit CPIP postoperatively. In these patients the mean score was 2.48 ± 0.22 compared to 2.06 ± 0.06 in patients that did not develop CPIP (Fig. 3). This means that patients developing CPIP had higher doses of weak or strong opioids or antineuropathic medication. Therefore, for the CPIP patients the duration of the stay increased from 1.97 *d* ± 1.61 *d* to 2.5 *d* ± 1.85 *d*, since the analgesic medication was increased in a step up approach. However, most of the adjustments were made in the first 24 h after surgery, so the increase in the duration of stay was not significant (*p* = 0.053).

## Discussion

In summary, we identified several predictors for CPIP in our patient cohort. Based on our data, younger patient age and a higher analgesic score have a significant influence to the development of CPIP.

Inguinal hernia repair represents a common surgical procedure. Minimal-invasive and open surgery are used to treat inguinal hernias. Although these surgical procedures are associated with only few risks, CPIP is a common complication after inguinal hernia surgery. However, while some predictors of CPIP have been described [8, 16], robust evidence about the pathogenesis of this pain and the pain resolution are still not investigated. Here, we performed a retrospective analysis of real-world data in our non-selective cohort to investigate possible predictors for the development of CPIP comparing the subgroups depending on the surgical technique. We examined outcomes of patients receiving either Lichtenstein, TEP or TAPP. The study population reflects the current state of care in clinical practice in a tertiary center. There were significant differences in between the patient population receiving Lichtenstein, TEP or TAPP. Based on our data, these differences correspond to the indications for each surgical procedure [1]. An open surgical technique is preferred in multimorbid, patients, patients with prior abdominal surgery, or patients with anticoagulation. The TEP is performed almost exclusively in male patients without many co-morbidities. TAPP is the method of choice in women, as a possible femoral hernias can be detected. Also, the opposite side can be assessed during the operation. Similarly, younger patients were more likely to undergo a minimal-invasive surgical technique. his distribution of patients among the predominant surgical techniques is also shown by previous studies [17, 18]. These distribution might explain why we observed no difference in CPIP between the three surgical procedures in our study. Yet, there is plenty of evidence in the literature, that minimal invasive procedures led to a reduction in CPIP due to minimal access trauma and due to the fact that the inguinal nerves remain in their natural embedding [8].

In further subgroup analysis we compared the two groups “no pain” and “CPIP”. Similar to previous studies, our data showed patients with CPIP are younger. Unlike previous studies we did not observe significant differences between the two groups with previous chronic pain and smaller hernia defects [19–22]. Accordingly, the differentiated indication for surgery is of absolutely crucial importance. [1, 19].

We also introduced a new analgesics score. Pain is recorded using the V/NRS as standard. Perioperative analgesics are administered until the patient is pain-free (pain <  = 3) or does not demand more medication. However, the perioperative use of analgesics influences the pain level. V/NRS only measures pain without considering the amount and potency of analgesics used. Based on our data an increased analgesic use can be a possible predictor for the development of CPIP. Patients with CPIP showed a higher consumption of analgesics directly postoperatively. Since early treatment of this postoperative pain could help to reduce the percentage of CPIP [23], we suggest that patients with an analgesics score ≥3 (e.g., patients under opioid medication like tilidine plus non opioids) at discharge should be included in a follow up after 4–6 weeks. However, this suggestion has to be validated in a prospective study. Current studies implementing transitional pain services [24] will contribute to the questions which minimal measures are necessary for prevention of chronification. Interestingly, presurgical chronic pain and pain medication were not a predictor as seen in other studies before [25]. It is possible that presurgical pain might be relevant for certain types of surgeries.

In the past many different factors have been identified to reduce CPIP. Sophisticated studies suggested that mechanical mesh fixation should be avoided and proper knowledge of inguinal nerves is essential [26]. Another possible risk factor that should be considered is the use of different mesh types (e.g., weight, pore size, tensile strength, and elasticity). Short-term follow-up studies have compared heavyweight mesh (HWM) and lightweight mesh (LWM). It was shown that LWM can lead to a lower incidence of CPIP and foreign body sensation during Lichtenstein surgery [27–30]. In contrast, medium- and long-term studies showed no differences in the development of chronic pain with the use of LWM or HWM [31].

Although our data show interesting results on the prediction of CPIP based on postoperative analgesic consumption, many questions remain. While probably precise surgical technique and proper nerve management can reduce neuropathic pain, the problem of nociceptive pain remains. Two factors may play a role here: the genetic nature of the metabolism of analgesics and the genetic nature of pain sensitivity as such [28–34]. Further studies will be needed here. Also, whether newer surgical procedures such as robotic-assisted TAPP can improve postoperative quality of life outcomes remains to be seen [35].

## Conclusion

The aim of the study was to identify predictors for the development of CPIP in our patients and generate a tool that enables early identification of potential patients with a higher risk for CPIP. In summary, there are several predictors for CPIP. Based on our data, younger patient age and a higher analgesic score have a significant influence. This score might help to improve the identification of possible CPIP patients. However, further prospective studies are necessary to better understand the pathogenesis of CPIP, to confirm these predictors and to establish new therapeutic approaches.
